# Supplementary material for: Refining the diagnostic approach to latent tuberculosis Infection with Quantiferon gold plus: A retrospective analysis of borderline results
Source: PLoS One. 2025 Sep 8;20(9):e0330345. doi: 10.1371/journal.pone.0330345 (PMC12416684; doi:10.1371/journal.pone.0330345)
Supplement: S1 Table — (DOCX) [file pone.0330345.s001.docx]

| **Table S1. Mean age and categorical distribution of follow-up QFT results when retesting those with initial results in the borderline range (0.20–0.70 IU/ml), by continent.** | | | | | | | |
| --- | --- | --- | --- | --- | --- | --- | --- |
| **Initial QFT**  **result by continent** | | **Results of follow-up QFT test* N (%)** | | | | | |
|  |  | **Indeterminate** | **Negative** | **BL Negative** | **BL Positive** | **Positive** | **Total** |
| **BL Negative** | **Africa** |  | 2 (3.1) |  | 1 (1.6) | 1 (1.6) | 4 (6.3) |
|  | Age (Mean years) |  | 51.4 |  | 54.6 | 46.2 | 50.9 |
|  | **Unknown** |  | 1 (1.6) |  |  | 1 (1.6) | 2 (3.1) |
|  | Age (Mean years) |  | 56.1 |  |  | 35.6 | 45.9 |
|  | **Europe** | 3 (4.7) | 18 (28.1) | 4 (6.3) | 1 (1.6) | 1 (1.6) | 27 (42.2) |
|  | Age (Mean years) | 52.1 | 49.9 | 65.7 | 66.1 | 73.4 | 53.9 |
|  | **South America** |  | 6 (9.4) |  | 1 (1.6) | 1 (1.6) | 8 (12.5) |
|  | Age (Mean years) |  | 51.6 |  | 11.5 | 54.0 | 46.9 |
|  | **Total** | 3 (4.7) | 27 (42.2) | 4 (6.3) | 3 (4.7) | 4 (6.3) | 41 (64.1) |
|  | Age (Mean years) | 52.1 | 50.6 | 65.7 | 44.1 | 52.3 | 51.9 |
| **BL Positive** | **Africa** |  | 2 (3.1) |  | 1 (1.6) | 1 (1.6) | 4 (6.3) |
|  | Age (Mean years) |  | 68.3 |  | 61.2 | 71.3 | 67.3 |
|  | **Unknown** |  | 1 (1.6) |  | 1 (1.6) | 1 (1.6) | 3 (4.7) |
|  | Age (Mean years) |  | 28.7 |  | 46.7 | 65.4 | 47.0 |
|  | **Europe** |  | 8 (12.5) | 5 (7.8) | 1 (1.6) | 1 (1.6) | 15 (23.4) |
|  | Age (Mean years) |  | 49.6 | 46.4 | 52.6 | 63.2 | 49.6 |
|  | **South America** |  | 1 (1.6) |  |  |  | 1 (1.6) |
|  | Age (Mean years) |  | 55.9 |  |  |  | 55.9 |
|  | **Total** |  | 12 (18.8) | 5 (7.8) | 3 (4.7) | 3 (4.7) | 23 (35.9) |
|  | Age (Mean years) |  | 51.5 | 46.4 | 53.5 | 66.6 | 52.6 |
| **Total BL** | **Africa** |  | 4 (6.3) |  | 2 (3.1) | 2 (3.1) | 8 (12.5) |
|  | Age (Mean years) |  | 59.9 |  | 57.9 | 58.7 | 59.1 |
|  | **Unknown** |  | 2 (3.1) |  | 1 (1.6) | 2 (3.1) | 5 (7.8) |
|  | Age (Mean years) |  | 42.4 |  | 46.7 | 50.5 | 46.5 |
|  | **Europe** | 3 (4.7) | 26 (40.6) | 9 (14.1) | 2 (3.1) | 2 (3.1) | 42 (65.6) |
|  | Age (Mean years) | 52.1 | 49.8 | 55.0 | 59.4 | 68.3 | 52.4 |
|  | **South America** |  | 7 (10.9) |  | 1 (1.6) | 1 (1.6) | 9 (14.1) |
|  | Age (Mean years) |  | 52.2 |  | 11.5 | 54.0 | 47.9 |
|  | **Total** | 3 (4.7) | 39 (60.9) | 9 (14.1) | 6 (9.4) | 7 (10.9) | 64 (100.00) |
|  | Age (Mean years) | 52.1 | 50.9 | 55.0 | 48.8 | 58.5 | 52.1 |
| Note. **BL:** borderline. * Negative: <0.2 IU/mL; borderline negative: ≥0.2 and <0.35 IU/mL; borderline positive: 0.35-0.7 IU/mL; positive: >0.7 IU/mL. | | | | | | | |
