# Supplementary material for: Refining the diagnostic approach to latent tuberculosis Infection with Quantiferon gold plus: A retrospective analysis of borderline results
Source: PLoS One. 2025 Sep 8;20(9):e0330345. doi: 10.1371/journal.pone.0330345 (PMC12416684; doi:10.1371/journal.pone.0330345)
Supplement: Attachment — Submitted filename: F_Response to reviewers and editor.docx [file pone.0330345.s002.docx]

**Author´s Response to editor and reviewers:**

Dear editor and reviewers,

We appreciate very much that you have considered our work.

Please, find enclosed our Revision to Manuscript number: PONE-D-24-52042”

As requested, we include in the uploaded files:

1. Response to reviewers.

2. Revised Manuscript with Track Changes.

3. Unmarked version of the revised paper.

We are grateful to the reviewers and editor for their valuable comments, which have been reviewed in depth and added to the manuscript when required. We hope this revised version of the manuscript meets the criteria required to be published in PLOS One.

Please, find the changes introduced in the text highlighted and indicated point by point in the letter of response attached below. We hope these changes and documents attached would be helpful for the revision process.

All authors have contributed to, seen, and approved the final revised version of the manuscript. They have given necessary attention to ensure the integrity of the work.

If there are any questions or problems with this revision, please do not hesitate to contact me: Paula López Roa, Clinical Microbiology and Parasitology Department, Hospital Universitario 12 de Octubre, Madrid, Spain. Av. de Córdoba, s/n, 28041 Madrid, Spain; e-mail: plroa@salud.madrid.org

Yours sincerely,

Paula López Roa on behalf of all the co-authors.

**Author´s response to reviewers:**

**Reviewer #1:**

**Congratulations for submitting this manuscript and for completing this study as part of the body of knowledge in the field of TB diagnosis. Generally, the paper is clear and straightforward in addressing the research objective.**

**Reviewer comment 1: In the methods section, you may specify the general objective you mentioned in the introduction.**

**Response:** Thank you for your suggestion. We have addressed this by adding the following sentence to the Methods section “We aimed to assess the proportion of QFT tests yielding equivocal results and to evaluate the outcomes of retesting these cases by establishing a defined borderline range”

**Reviewer comment 2: For instance, you could have explained more why did you choose the demographic variables and further describe what could be its implications when you include it in your study as far as QFT is concerned.**

**Response:** Thank you for your comment. We agree that clarifying the importance of the selected demographic variables is relevant. To address this, we have added the following sentence to the Methods section: “These variables have been associated, along with borderline test results, with both the likelihood of conversions and reversions of Interferon γ Release Assays [1].”

**Reviewer comment 3: In the resuls section, please be consistent with the use of proper formatting of tables based on the guidelines of the PLOS.**

**Response:** Thank you for your feedback. We have reviewed and updated the formatting of the tables in the Results section to ensure they are consistent with the PLOS guidelines.

**Reviewer comment 4: Another suggestion would be to include contrasting studies (aside from consistent ones), whenever available. Additional references in the discussion section further improves the credibility of the findings. Lastly, you have a good conclusion. May you keep on working on this kind of studies that would possibly inform future policy. God bless.**

**Response:** Thank you for your valuable suggestion. We have include contrasting studies and expanded the discussion section with additional references. This has improved the contextualisation and overall understanding of the article.

“Interpreting diagnostic test results requires careful consideration of the clinical and epidemiological context. The risk of developing active TB varies among individuals with positive IGRA reactions. Studies have demonstrated a **correlation** between IGRA result magnitude and the incidence of active TB, i**ncluding cases within** the borderline range, **suggesting a dose-response relationship [2].** However, in regions with low TB prevalence, individuals presenting with borderline QFT results often experience reversion to negative upon retesting. A study involving 58,539 subjects in a low-endemicity country found that among those with initial borderline results who were retested, **38% reverted to negative, with no cases of incident active TB reported within two years.** This finding suggests that **a significant proportion of initial borderline results may represent false positives** [3]. Consequently, the risk of missing a true LTBI in such cases is relatively low, and avoiding unnecessary treatment minimizes the potential for adverse effects and disruptions to daily life.

Conversely, in populations at higher risk for TB progression, such as immunosuppressed individuals, the emphasis shifts toward maximizing test sensitivity, even at the expense of specificity. Failure to diagnose and treat LTBI in these patients can lead to severe outcomes, including the development of active TB disease. Therefore, integrating other risk factors—such as recent exposure to active TB cases, underlying comorbidities, and the degree of immunosuppression—along with underlying determinants such as access to healthcare, socioeconomic status, and level of vulnerability is essential in guiding clinical decisions [4].

Contrasting evidence suggests that most borderline QFT results reflect true antigen-specific responses rather than random variability, and that the presence of various non-IGRA risk factors and parameters of TB infection showed a gradient along increasing quantitative QFT results [5, 6]. This study provided interesting data on QFT results, but it is important to note that the study was conducted within the context of a contact study, and not in other settings, where the interpretation of the results and the risk of progression to active TB may vary significantly. We agree with Wikell et al. [3] that the inherent variability of the technique should be given due consideration, and that further study is required to ascertain the clinical significance of borderline results in terms of the development of active TB in a range of clinical settings.”

**Reviewer #2**

**This retrospective review study aims to assess the (1) clinical characteristics of those with borderline tests and (2) the clinical applicability of borderline QFT-gold test results by analyzing the results of repeat testing (ie conversions/reversions) for the 7-8% of patients with borderline results on the initial test. Given the number of patients in the borderline category to reverted or converted, this study highlights the challenge of interpreting single borderline results and argues for repeat testing over time. This study did demonstrate that the risk of progressing to active disease in the borderline group (~5%) was different from that of both the negative (~0.1%) and positive group (~5%), though it should be noted that this study is retrospective in nature and sample numbers are small in the borderline group.**

**Reviewer comment 1. Did this study include children? It was not clear from the Methods section and given the emphasis on age as a correlate of borderline QFT results, I would recommend being explicit about the age range included in the study, especially if those <5yo are included.**

**Response:** We agree that clarifying the age range is important. We have included this information in both the Methods and Results sections:

“The study was conducted in a routine clinical setting at a tertiary hospital in Madrid, focusing on patients of all age groups who underwent QFT testing. Children were not excluded, as previous research suggests that the QFT assay is accurate in pediatric practice, with good sensitivity [7]”

“The study population comprised individuals aged from 0.05 to 98.52 years, with a mean age of 51.64 years (SD = 19.24). Focusing on younger subgroups, individuals under 18 years of age accounted for 4.7% (466 patients), while those under 5 years represented only 1.2% (122 patients) of the total population.”

**Reviewer comment 2. Only 64/758 borderline patients were retested, but the clinical characteristics of this subset are not included in the study nor is the re-testing interval. While not all data may be available, it would be helpful to know if these patients had risk factors or other clinical characteristics different from the overall cohort that prompted the closer follow-up (for example, heavier exposure to TB, country of origin, symptoms of active TB, etc) compared to those that were not re-tested? Additionally, is it confirmed whether these 64 patients are treatment-naive given treatment data was not included as part of the study?**

**Response:** Thank you for your valuable comment. Unfortunately, due to the retrospective nature of the study, we were unable to obtain data on the patients' risk factors, symptoms, or comorbidities. To address the time interval between the initial testing and the repeated testing, we have added the "Months until retesting Mean(SD)" and "Range" columns to Table 2. Additionally, we have included Supplementary Table 1, where the data for continent and mean age can be found in detail. We hope these additions provide greater clarity on the patients' demographics. Moreover, all individual-level data for each patient and result will be made publicly available in a repository.

**Reviewer comment 3. While re-testing of borderline QFT results may provide additional diagnostic clarity, retesting ~8% of patients is an additional health care cost and risks losing patients to follow-up, some of whom would go on to develop active TB, but has the advantage of potentially avoiding unnecessary LTBI treatment in a subset of individuals. It may be helpful to include in the discussion potential positive and negative consequences of initiating this intervention.**

**Reviewer comment 4. It would also be helpful to include in the discussion the effects of serial testing on public health in both low endemicity and high endemicity settings.**

**Response to comments 3 and 4:** Thank you for your valuable contributions. We have added some paragraphs addressing these topics in the discussion section:

“Interpreting diagnostic test results requires careful consideration of the clinical and epidemiological context. The risk of developing active TB varies among individuals with positive IGRA reactions. Studies have demonstrated a **correlation** between IGRA result magnitude and the incidence of active TB, i**ncluding cases within** the borderline range, **suggesting a dose-response relationship [2].** However, in regions with low TB prevalence, individuals presenting with borderline QFT results often experience reversion to negative upon retesting. A study involving 58,539 subjects in a low-endemicity country found that among those with initial borderline results who were retested, **38% reverted to negative, with no cases of incident active TB reported within two years.** This finding suggests that **a significant proportion of initial borderline results may represent false positives** [3]. Consequently, the risk of missing a true LTBI in such cases is relatively low, and avoiding unnecessary treatment minimizes the potential for adverse effects and disruptions to daily life.

Conversely, in populations at higher risk for TB progression, such as immunosuppressed individuals, the emphasis shifts toward maximizing test sensitivity, even at the expense of specificity. Failure to diagnose and treat LTBI in these patients can lead to severe outcomes, including the development of active TB disease. Therefore, integrating other risk factors—such as recent exposure to active TB cases, underlying comorbidities, and the degree of immunosuppression—along with underlying determinants such as access to healthcare, socioeconomic status, and level of vulnerability is essential in guiding clinical decisions [4].

Contrasting evidence suggests that most borderline QFT results reflect true antigen-specific responses rather than random variability, and that the presence of various non-IGRA risk factors and parameters of TB infection showed a gradient along increasing quantitative QFT results [5, 6]. This study provided interesting data on QFT results, but it is important to note that the study was conducted within the context of a contact study, and not in other settings, where the interpretation of the results and the risk of progression to active TB may vary significantly. We agree with Wikell et al. [3] that the inherent variability of the technique should be given due consideration, and that further study is required to ascertain the clinical significance of borderline results in terms of the development of active TB in a range of clinical settings.

Furthermore, given the proportion of borderline QFT results observed in our study and the low incidence of TB progression within this subgroup, one could argue that clinical follow-up alone may suffice, with treatment initiated as necessary. However, refining diagnostic classification strategies would enhance the efficient allocation of healthcare resources and could also reduce the stigma associated with a positive diagnosis. Targeting preventive treatment to individuals most likely to benefit not only optimizes cost-effectiveness but also reduces unnecessary interventions in those at low risk for disease progression.

From a public health perspective, any modifications to TB screening protocols must consider the availability of resources—both material and personnel—to ensure appropriate patient follow-up. Implementation strategies should be tailored to local epidemiological conditions and healthcare infrastructure to maximize feasibility and impact.

Given the significant morbidity and mortality associated with TB, further research is essential to determine which individuals with IGRA conversion or reversion are most likely to benefit from TB preventive treatment, and more studies are warranted across diverse geographical regions and ethnic populations. A deeper understanding of the clinical significance of borderline results for specific patient populations will be crucial in refining LTBI management strategies and improving patient outcomes.”

And we also have added a brief statement in the conclusions of the study:

“While repeat testing offers valuable insights, it may not always be feasible, and further studies are needed to assess the long-term clinical outcomes of borderline QFT results.”

**Reviewer #3:**

**The manuscript entitled "Refining the Diagnostic Approach to Latent Tuberculosis Infection with Quantiferon Gold Plus: A Retrospective Analysis of Borderline Results" needs these revisions**

**Reviewer comment 1: Please check keywords according to MESH browser; delete QuantiFERON-TB Gold Plus and borderline results from keywords.**

**Response:** Thank you for your valuable comment. We have revised the keywords and have replaced "QuantiFERON-TB Gold Plus" and "borderline results" with more appropriate terms according to the MESH browser.

**Reviewer comment 2: Please write about the age range of patients**

**Response:** We agree that clarifying the age range is important. We have added this information in results section:

“The study population comprised individuals aged from 0.05 to 98.52 years, with a mean age of 51.64 years (SD = 19.24). Focusing on younger subgroups, individuals under 18 years of age accounted for 4.7% (466 patients), while those under 5 years represented only 1.2% (122 patients) of the total population.”

**Reviewer comment 3: What are the rules for repeat testing, what is the minimum time interval for repeat testing**.

**Response:** Thank you, we agree with your suggestion and have expanded on this topic in the Methods section:

“Patients were tested based on clinical indications, such as suspected latent or active tuberculosis, or for screening purposes. Repeat testing was carried out when deemed necessary by the attending physicians, in consultation with microbiologists. This occurred in cases where there were inconsistencies with expected results, a high clinical suspicion of TB, or indeterminate results.”

To clarify the time intervals for repeat testing, we have included two new columns in Table 2: 'Months until retesting (Mean ± SD)' and 'Range'. This addition provides a clear overview of the minimum time intervals and variability between initial and repeated testing.

**Reviewer #4:**

**The manuscript is well written and highlighted the important subject of diagnosing LTBI, and more importantly to the borderline outcomes interrupting the results which alters in the retesting. Applause to the authors for extracting and analysing such data to develop this manuscript. There are a couple of minor comments/suggestions to this.**

**Reviewer comment 1: Line 75 - 86: this section could explore further the setup of health facility which the study was conducted, such as TB facility or any other, how patients were selected to test, QTF testing criteria, criteria for retesting of borderline results, and treatment and follow up or further care after LTBI diagnosis.**

**Response:** Thank you for your valuable comment. We agree with your suggestion and have expanded on this topic in the Methods section. The following additional information has been included:

**“**A retrospective review of patients with QFT results between 2019 and 2023 was conducted, including demographic data such as age, gender, and country of birth. These variables have been associated, along with borderline test results, with both the likelihood of conversions and reversions of Interferon γ Release Assays [1]. The study was conducted in a routine clinical setting at a tertiary hospital in Madrid, focusing on patients of all age groups who underwent QFT testing. Children were not excluded, as previous research suggests that the QFT assay is accurate in pediatric practice, with good sensitivity [7]. The hospital is a 1,300-bed acute teaching hospital that serves as a referral centre for a population of 462,000 [8], of whom 33.6% are foreign-born [9]. The population is comprised of 69% individuals between the ages of 14 and 64 years, 11% between 65 and 79 years, and 6% over 80 years. The incidence of tuberculosis in the population of the three health districts attached to the hospital is among the highest in the Community of Madrid, ranging from almost 19 cases per 100,000 inhabitants in the districts of Usera or Villaverde, to 14.8 per 100,000 in Carabanchel, compared to the Community average of 8.59 per 100,000 [10].

Data for research purposes were accessed from January 2024 to May 2024. Patients were tested based on clinical indications, such as suspected latent or active tuberculosis, or for screening purposes. Repeat testing was carried out when deemed necessary by the attending physicians, in consultation with microbiologists. This occurred in cases where there were inconsistencies with expected results, a high clinical suspicion of TB, or indeterminate results.”

Unfortunately, due to the retrospective nature of the study, data on the initiation of treatment and follow up after LTBI diagnosis could not be included.

**Reviewer comment 2: The demographic variables are only age, gender and country of birth. Are there any other variables included in the regression model such as previous TB history, HIV coinfection, or other comorbidities? It will be interesting to see the relation with such variables. Or are they not available as a limitation of the retrospective study.**

Thank you for your insightful comment. Unfortunately, due to the retrospective nature of the study and the large number of patients included, we do not have access to additional data on variables such as previous TB history, HIV coinfection, or other comorbidities. These variables were not consistently recorded and thus could not be included in the regression model. We acknowledge this as a limitation of the study.

**Reviewer comment 3: Line 188 - 124: this is an interesting outcome after repeated testing. What is the time interval between initial testing vs repeated testing?**

**Response:** Thank you for your valuable comment. We have added the "Months until retesting Mean(SD)" and "Range" columns to Table 2 to address the time interval between initial testing and repeated testing. Additionally, we have included Supplementary Table 1, where the data for each individual patient can be observed in detail. We hope this provides more clarity on the time intervals for repeated testing.

**Reviewer #5:**

**Reviewer comment 1: There is a very small percentage of patients who fall within the borderline range . Of these your classification improves the predictability of the classification. However only 1.1% of the cases develop TB during followup. It may not be too difficult to just follow-up those who fall in the border line range. So what is the added value of your improved classification.**

We appreciate the reviewer’s comment and agree that the proportion of patients with borderline QFT results is small, and the risk of progression to active TB in this subgroup is low (1.1%). To clarify, our classification aims to address the uncertainty in managing borderline results by distinguishing between those who may benefit from preventive treatment and those who can be safely monitored through clinical follow-up alone.

Regarding the reviewer’s question about follow-up, we propose that the focus should be on all individuals with initial borderline results (n=64), as retesting can help identify those who revert to negative and are unlikely to progress to active TB. However, the added value of our improved classification lies in its ability to refine risk stratification and guide clinical decisions more effectively.

We have added the following sentence in the manuscript:

“Furthermore, given the proportion of borderline QFT results observed in our study and the low incidence of TB progression within this subgroup, one could argue that clinical follow-up alone may suffice, with treatment initiated as necessary. However, refining diagnostic classification strategies would enhance the efficient allocation of healthcare resources and could also reduce the stigma associated with a positive diagnosis. Targeting preventive treatment to individuals most likely to benefit not only optimizes cost-effectiveness but also reduces unnecessary interventions in those at low risk for disease progression.”

**Reviewer comment 2: You have said that the Percentages are more in the countries like Africa? Why is this so? Would the prevalence of NTM have something to do with it? This point could be addressed more in the discussion.**

**Response:** Thank you for your helpful suggestion. We agree that the higher percentages of borderline QFT results observed in regions such as Africa and South America could potentially be influenced by the prevalence of non-tuberculous mycobacteria (NTM). However, it is important to highlight that since NTM reporting is not mandatory, comprehensive data regarding the prevalence of these mycobacteria across different continents is very limited. As a result, it is difficult to draw any definitive conclusions on whether this factor contributes to the observed differences in borderline percentages.

Considering this, we have added the following paragraph in the discussion section of the manuscript:

“The higher percentages of borderline QFT results observed in Africa and South America may be linked to the higher prevalence of TB in these regions. While this is a plausible explanation, it remains speculative, as TB prevalence was not included in our regression model. It is also important to note that certain species of non-tuberculous mycobacteria (NTM), such as *M. kansasii*, *M. marinum*, and *M. szulgai*, can cause positive QFT results [11]. However, due to the limited availability of comprehensive data on the prevalence of these NTMs across different continents, it is not possible to determine whether this factor influences the observed differences in borderline percentages. Nonetheless, regardless of the underlying cause, there appears to be added value or greater indication for retesting borderline results in these populations.”

**Reviewer #6:**

**Reviewer comment 1: In the introduction, it would be beneficial for the authors to highlight any existing studies that have evaluated and compared the results between QFT-Plus and QFT-GIT. Discussing any observed differences, such as costs and immune responses, would provide valuable insights, and including relevant references would enhance the credibility of the review.**

**Response:** thank you for your suggestion**.** We have incorporated this information into the introduction section:

“Compared to its predecessor, the QuantiFERON-TB Gold In-Tube (QFT-GIT), the QFT-Plus has demonstrated improved sensitivity [12], while maintaining comparable or, in some studies, improved specificity [13, 14]. Additionally, QFT-Plus may offer greater sensitivity for predicting active TB [15]. However, IGRA tests are more expensive than the tuberculin skin test (TST), which may remain a cost-effective option in some high TB burden countries. Factors such as cost-effectiveness and local epidemiological conditions, including TB prevalence and resource availability, are critical when choosing between these tests [14]. In our clinical practice, we have adopted the QFT-Plus as the primary diagnostic tool for LTBI.”

**Reviewer comment 3: Furthermore, it is critical to emphasize that clinicians are not just interested in laboratory evaluation ranges, but in how these ranges are defined by population stratification, particularly by age. For instance, in Europe, the age demographic of the Caucasian population may differ. More importantly, the patient's overall health status plays a pivotal role in interpreting laboratory data. A holistic approach to clinical management should involve evaluating the population based on race and understanding the type of immune response, factoring in essential epidemiological and clinical variables such as cancer, autoimmune disorders, protein intake, BMI, coinfections, and access to healthcare, which can differ across countries.**

Thank you for your valuable comment. We acknowledge the importance of considering population stratification, particularly by age, in the interpretation of laboratory evaluation ranges. As you correctly pointed out, factors such as race, immune response variability, and key epidemiological and clinical aspects (e.g., cancer, autoimmune disorders, protein intake, BMI, coinfections, and healthcare access) can significantly influence laboratory values and clinical outcomes. However, due to the retrospective nature of our study, we recognize certain limitations in addressing these variables comprehensively. While our dataset does not allow for a detailed stratification based on all these factors, we will highlight this limitation in the discussion section and incorporate relevant references to support the broader clinical context you have mentioned.

In the discussion section we have emphasized the need of this important clinical approach:

“ Interpreting diagnostic test results requires careful consideration of the clinical and epidemiological context. The risk of developing active TB varies among individuals with positive IGRA reactions. Studies have demonstrated a **correlation** between IGRA result magnitude and the incidence of active TB, i**ncluding cases within** the borderline range, **suggesting a dose-response relationship [2].** However, in regions with low TB prevalence, individuals presenting with borderline QFT results often experience reversion to negative upon retesting. A study involving 58,539 subjects in a low-endemicity country found that among those with initial borderline results who were retested, **38% reverted to negative, with no cases of incident active TB reported within two years.** This finding suggests that **a significant proportion of initial borderline results may represent false positives** [3]. Consequently, the risk of missing a true LTBI in such cases is relatively low, and avoiding unnecessary treatment minimizes the potential for adverse effects and disruptions to daily life.

Conversely, in populations at higher risk for TB progression, such as immunosuppressed individuals, the emphasis shifts toward maximizing test sensitivity, even at the expense of specificity. Failure to diagnose and treat LTBI in these patients can lead to severe outcomes, including the development of active TB disease. Therefore, integrating other risk factors—such as recent exposure to active TB cases, underlying comorbidities, and the degree of immunosuppression—along with underlying determinants such as access to healthcare, socioeconomic status, and level of vulnerability is essential in guiding clinical decisions [4].

Contrasting evidence suggests that most borderline QFT results reflect true antigen-specific responses rather than random variability, and that the presence of various non-IGRA risk factors and parameters of TB infection showed a gradient along increasing quantitative QFT results [5, 6]. This study provided interesting data on QFT results, but it is important to note that the study was conducted within the context of a contact study, and not in other settings, where the interpretation of the results and the risk of progression to active TB may vary significantly. We agree with Wikell et al. [3] that the inherent variability of the technique should be given due consideration, and that further study is required to ascertain the clinical significance of borderline results in terms of the development of active TB in a range of clinical settings.

Furthermore, given the proportion of borderline QFT results observed in our study and the low incidence of TB progression within this subgroup, one could argue that clinical follow-up alone may suffice, with treatment initiated as necessary. However, refining diagnostic classification strategies would enhance the efficient allocation of healthcare resources and could also reduce the stigma associated with a positive diagnosis. Targeting preventive treatment to individuals most likely to benefit not only optimizes cost-effectiveness but also reduces unnecessary interventions in those at low risk for disease progression.

From a public health perspective, any modifications to TB screening protocols must consider the availability of resources—both material and personnel—to ensure appropriate patient follow-up. Implementation strategies should be tailored to local epidemiological conditions and healthcare infrastructure to maximize feasibility and impact.

Given the significant morbidity and mortality associated with TB, further research is essential to determine which individuals with IGRA conversion or reversion are most likely to benefit from TB preventive treatment, and more studies are warranted across diverse geographical regions and ethnic populations. A deeper understanding of the clinical significance of borderline results for specific patient populations will be crucial in refining LTBI management strategies and improving patient outcomes. “

Thank you again for your constructive feedback.

1.	Wang MS, Li-Hunnam J, Chen YL, Gilmour B, Alene KA, Zhang YA, et al. Conversion or reversion of interferon gamma release assays for Mycobacterium tuberculosis infection: a systematic review and meta-analysis. Clin Infect Dis. 2024. Epub 20240702. doi: 10.1093/cid/ciae357. PubMed PMID: 38954503.

2.	Ledesma JR, Ma J, Zheng P, Ross JM, Vos T, Kyu HH. Interferon-gamma release assay levels and risk of progression to active tuberculosis: a systematic review and dose-response meta-regression analysis. BMC Infectious Diseases. 2021;21(1). doi: 10.1186/s12879-021-06141-4.

3.	Wikell A, Jonsson J, Dyrdak R, Henningsson AJ, Eringfält A, Kjerstadius T, et al. The Impact of Borderline Quantiferon-TB Gold Plus Results for Latent Tuberculosis Screening under Routine Conditions in a Low-Endemicity Setting. Journal of Clinical Microbiology. 2021;59(12). doi: 10.1128/jcm.01370-21.

4.	Lönnroth K, Migliori GB, Abubakar I, D'Ambrosio L, de Vries G, Diel R, et al. Towards tuberculosis elimination: an action framework for low-incidence countries. Eur Respir J. 2015;45(4):928-52. doi: 10.1183/09031936.00214014. PubMed PMID: 25792630; PubMed Central PMCID: PMCPMC4391660.

5.	Uzorka JW, Kroft LJM, Bakker JA, van Zwet EW, Huisman E, Knetsch-Prins C, et al. Proof of concept that most borderline Quantiferon results are true antigen-specific responses. Eur Respir J. 2017;50(5). Epub 20171116. doi: 10.1183/13993003.01630-2017. PubMed PMID: 29146607.

6.	Uzorka JW, Bossink AWJ, Franken WPJ, Thijsen SFT, Leyten EMS, van Haeften AC, et al. Borderline QuantiFERON results and the distinction between specific responses and test variability. Tuberculosis (Edinb). 2018;111:102-8. Epub 20180607. doi: 10.1016/j.tube.2018.06.002. PubMed PMID: 30029893.

7.	Buonsenso D, Delogu G, Perricone C, Grossi R, Careddu A, De Maio F, et al. Accuracy of QuantiFERON-TB Gold Plus Test for Diagnosis of Mycobacterium tuberculosis Infection in Children. Journal of Clinical Microbiology. 2020;58(6). doi: 10.1128/jcm.00272-20.

8.	“12 de Octubre” University Hospital activity report, 2023. Servicio Madrileño de Salud, Department of Health, Community of Madrid. Available at: https://www.comunidad.madrid/hospital/12octubre/nosotros/memorias. Accessed: 17 March 2025.

9.	Community of Madrid Statistical Institute. Annual census 2022. Available at: https:\\www.madrid.org\iestadis\fijas\estructu\demograficas\padron\pc22ext.htm. Accessed: 17 March 2025.

10.	Regional Register of Tuberculosis Cases of the Community of Madrid. Report for the year 2023. Epidemiological Bulletin of the Community of Madrid. Number 6. Volume 29. June 2024. Available at: https://www.comunidad.madrid/servicios/salud/tuberculosis. Accessed: 17 March 2025.

11.	QIAGEN. QuantiFERON-TB Gold Plus (QFT-Plus) Package Insert [Internet]. 2023 Jan Available from: https://www.qiagen.com [cited 02/18/2025].

12.	Sotgiu G, Saderi L, Petruccioli E, Aliberti S, Piana A, Petrone L, et al. QuantiFERON TB Gold Plus for the diagnosis of tuberculosis: a systematic review and meta-analysis. J Infect. 2019;79(5):444-53. Epub 20190829. doi: 10.1016/j.jinf.2019.08.018. PubMed PMID: 31473271.

13.	Pourakbari B, Mamishi S, Benvari S, Mahmoudi S. Comparison of the QuantiFERON-TB Gold Plus and QuantiFERON-TB Gold In-Tube interferon-gamma release assays: A systematic review and meta-analysis. Adv Med Sci. 2019;64(2):437-43. Epub 20191003. doi: 10.1016/j.advms.2019.09.001. PubMed PMID: 31586819.

14.	R A, D M, Kv L, Suriya VR, R K. Performance of Current Diagnostic Tools in Detecting Latent Tuberculosis Among Healthcare Workers: A Systematic Review. Cureus. 2024. doi: 10.7759/cureus.70621.

15.	Lee JK, Lee HW, Heo EY, Yim JJ, Kim DK. Comparison of QuantiFERON-TB Gold Plus and QuantiFERON-TB Gold In-Tube tests for patients with active and latent tuberculosis: A prospective cohort study. J Infect Chemother. 2021;27(12):1694-9. Epub 20210816. doi: 10.1016/j.jiac.2021.08.003. PubMed PMID: 34412980.
